# Brain Differences Between Men and Women: Evidence From Deep Learning

**DOI:** 10.3389/fnins.2019.00185

**Published:** 2019-03-08

**Authors:** Jiang Xin, Yaoxue Zhang, Yan Tang, Yuan Yang

**Affiliations:** ^1^School of Computer Science and Engineering, Central South University, Changsha, China; ^2^Department of Neurology, Xiangya Hospital, Central South University, Changsha, China; ^3^Department of Physical Therapy and Human Movement Sciences, Feinberg School of Medicine, Northwestern University, Chicago, IL, United States

**Keywords:** gender difference, deep learning, neural network, diffusion MRI, entropy

## Abstract

Do men and women have different brains? Previous neuroimage studies sought to answer this question based on morphological difference between specific brain regions, reporting unfortunately conflicting results. In the present study, we aim to use a deep learning technique to address this challenge based on a large open-access, diffusion MRI database recorded from 1,065 young healthy subjects, including 490 men and 575 women healthy subjects. Different from commonly used 2D Convolutional Neural Network (CNN), we proposed a 3D CNN method with a newly designed structure including three hidden layers in cascade with a linear layer and a terminal Softmax layer. The proposed 3D CNN was applied to the maps of factional anisotropy (FA) in the whole-brain as well as specific brain regions. The entropy measure was applied to the lowest-level image features extracted from the first hidden layer to examine the difference of brain structure complexity between men and women. The obtained results compared with the results from using the Support Vector Machine (SVM) and Tract-Based Spatial Statistics (TBSS). The proposed 3D CNN yielded a better classification result (93.3%) than the SVM (78.2%) on the whole-brain FA images, indicating gender-related differences likely exist in the whole-brain range. Moreover, high classification accuracies are also shown in several specific brain regions including the left precuneus, the left postcentral gyrus, the left cingulate gyrus, the right orbital gyrus of frontal lobe, and the left occipital thalamus in the gray matter, and middle cerebellum peduncle, genu of corpus callosum, the right anterior corona radiata, the right superior corona radiata and the left anterior limb of internal capsule in the while matter. This study provides a new insight into the structure difference between men and women, which highlights the importance of considering sex as a biological variable in brain research.

## Introduction

Recent studies indicate that gender may have a substantial influence on human cognitive functions, including emotion, memory, perception, etc., (Cahill, 2006). Men and women appear to have different ways to encode memories, sense emotions, recognize faces, solve certain problems, and make decisions. Since the brain controls cognition and behaviors, these gender-related functional differences may be associated with the gender-specific structure of the brain (Cosgrove et al., 2007).

Diffusion tensor imaging (DTI) is an effective tool for characterizing nerve fibers architecture. By computing fractional anisotropy (FA) parameters in DTI, the anisotropy of nerve fibers can be quantitatively evaluated (Lasi et al., 2014). Differences in FA values are thought to associate with developmental processes of axon caliber, myelination, and/or fiber organization of nerve fibers pathways. By computing FA, researchers has revealed subtle changes related to normal brain development (Westlye et al., 2009), learning (Golestani et al., 2006), and healthy aging (Kochunov et al., 2007). Nevertheless, existing studies are yet to provide consistent results on exploring the difference of brain structure between men and women. Ingalhalikar et al. (2014) argued that the men have greater intra-hemispheric connection via the corpus callosum while women have greater interhemispheric connectivity. However, other studies reported no significant gender difference in brain structure (Raz et al., 2001; Salat et al., 2005). A recent critical opinion article suggested that more research is needed to investigate whether men and women really have different brain structures (Joel and Tarrasch, 2014).

Most existing DTI studies used the group-level statistical methods such as Tract-Based Spatial Statistics (TBSS) (Thatcher et al., 2010; Mueller et al., 2011; Shiino et al., 2017). However, recent studies indicated that machine learning techniques may provide us with a more powerful tool for analyzing brain images (Shen et al., 2010; Lu et al., 2017; Tang et al., 2018). Especially, deep learning can extract non-linear network structure, realize approximation of complex function, characterize distributed representation of input data, and demonstrate the powerful ability to learn the essential features of datasets based on a small size of samples (Zeng et al., 2016, 2018a; Tian et al., 2018; Wen et al., 2018). In particular, the deep convolutional neural network (CNN) uses the convolution kernels to extract the features of image and can find the characteristic spatial difference in brain images, which may promise a better result than using other conventional machine learning and statistical methods (Cole et al., 2017).

In this study, we performed CNN-based analyses on the FA images and extracts the features of the hidden layers to investigate the difference between man and woman brains. Different from commonly used 2D CNN model, we innovatively proposed a 3D CNN model with a new structure including 3 hidden layers, a linear layer and a softmax layer. Each hidden layer is comprised of a convolutional layer, a batch normalization layer, an activation layer and followed by a pooling layer. This novel CNN model allows using the whole 3D brain image (i.e., DTI) as the input to the model. The linear layer between the hidden layers and the softmax layer reduces the number of parameters and therefore avoids over-fitting problems.

## Materials and Methods

### MRI Data Acquisition and Preprocessing

The database used in this work is from the Human Connectome Project (HCP) (Van Essen et al., 2013). This open-access database contains data from 1,065 subjects, including 490 men and 575 women. The ages range is from 22 to 36. This database represents a relatively large sample size compared to most neuroimaging studies. Using this open-access dataset allows replication and extension of this work by other researchers.

We performed DTI data preprocessing includes format conversion, b0 image extraction, brain extraction, eddy current correction, and tensor FA calculation. The first four steps were processed with the HCP diffusion pipeline, including diffusion weighting (bvals), direction (bvecs), time series, brain mask, a file (grad_dev.nii.gz) for gradient non-linearities during model fitting, and log files of EDDY processing. In the final step we use dtifit to calculate the tensors to get the FA, as well as mean diffusivity (MD), axial diffusivity (AD), and radial diffusivity (RD) values.

The original data were too large to train the model and it would cause RESOURCE EXAUSTED problem while training due to the insufficient of GPU memory. The GPU we used in the experiment is NVIDIAN TITAN_XP with 12G memory each. To solve the problem, we scaled the size of FA image to [58 × 70 × 58]. This procedure may lead to a better classification result, since a smaller size of the input image can provide a larger receptive field to the CNN model. In order to perform the image scaling, “dipy” (http://nipy.org/dipy/) was used to read the .nii data of FA. Then “ndimage” in the SciPy (http://www.scipy.org) was used to reduce the size of the data. Scaled data was written into the TFRecord files (http://www.tensorflow.org) with the corresponding labels. TFRecord file format is a simple record oriented binary format that is widely used in Tensorflow application for the training data to get a high performance of input efficiency. The labels were processed into the format of one-hot. We implemented a pipeline to read data asynchronously from TFRecord according to the interface specification provided by Tensorflow (Abadi et al., 2016). The pipeline included the reading of TFRecord files, data decoding, data type conversion, and reshape of data.

### CNN Model

We did the experiments on a GPU work station, which has four NVIDIA TITAN Xp GPUs. The operation system of the GPU work station was Ubutnu16.04. We used FSL to preprocess the data. The CNN model was designed using the open source machine learning framework Tensorflow (Abadi et al., 2016).

#### Model Design

**Figure 1:**
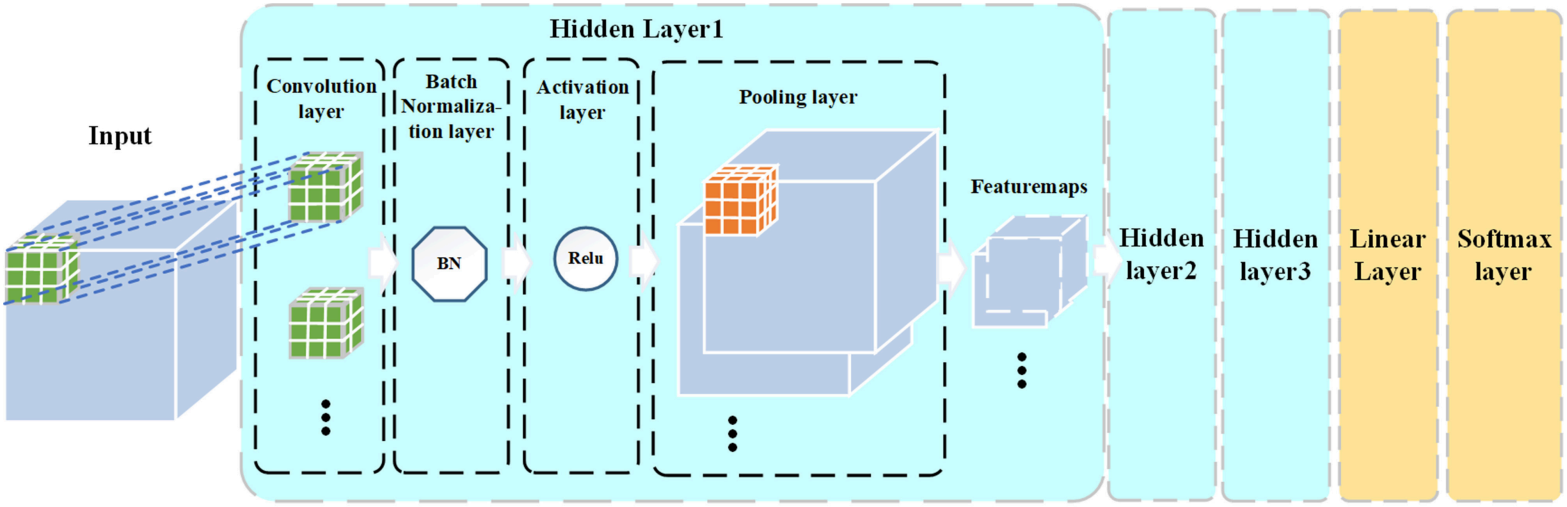
3D PCNN architecture.

The commonly used CNN structures are based on 2D images. When using a 2D CNN to process 3D MRI images, it needs to map the original image from different directions to get 2D images, which will lose the spatial structure information of the image. In this study, we designed a 3D CNN with 3D convolutional kernels, which allowed us to extract 3D structural features from FA images. Besides, traditional CNN model usually uses several fully connected layers to connect the hidden layers and the output layer. The fully connected layer may be prone to the over-fitting problem in binary classification when the number of samples is limited (like our data). To address this problem, we used a linear layer to replace the fully connected layer. The linear layer integrates the outputs of hidden layers (i.e., a 3D matrix comprised of multiple featuremaps) into the inputs (i.e., a 1D vector) of the output layer which is a softmax classifier. Moreover, we performed a Batch Normalization (Ioffe and Szegedy, 2015) after each convolution operation. The Batch Normalization is used to avoid internal covariate shift problem in training the CNN model. Therefore, our designed model is a 3D “pure” CNN (3D PCNN). The architecture of the 3D PCNN model is shown in Figure 1. The 3D PCNN consists of three hidden layers, a linear layer and a softmax layer. Each of the hidden layer contains a convolutional layer, a Batch Normalization layer, an activation layer, a pooling layer with several feature maps as the outputs.

##### Convolutional layer

The process of convolutional layer is to convolve the input vector *I* with the convolution kernel *K*, represented by *I*⊗*K*. The shape of the input vector in our 3D PCNN model was [*n, d, w, h, c*], where d, w, h, c represent the depth, width, height and channel numbers (which is 1 for a grayscale image) of the input vector, respectively, and n is the batch size which is a hyperparameter that was set to 45 (an empirical value) in this paper. In the first layer, the input size was 58 × 70 × 58 × 1, which was the 3D size (58 × 70 × 58) of the input image plus a single channel (grayscale image). The shape of the convolution kernel was [*d*_*k*_, *w*_*k*_, *h*_*k*_, *c*_*in*_, *c*_*out*_], where *d*_*k*_, *w*_*k*_, *h*_*k*_ represents the depth, width, and height of the convolution kernel, respectively. In all three hidden layers, the kernel size was set to3 × 3 × 3, which means that *d*_*k*_ = *w*_*k*_ = *h*_*k*_ = 3. The *c*_*in*_ is the number of input channels which is equal to the channel number of the input vector. The *c*_*out*_ is the number of output channels. As each kernel has an output channel, *c*_*out*_ is equal to the number of convolution kernels, and is also the same as the number of input channels for the next hidden layer. In all convolution layers, the moving stride of the kernel was set to 1 and padding mode was to “SAME.”

##### Batch normalization layer

Batch normalization was performed after the convolutional layer. Batch normalization is a kind of training trick which normalizes the data of each mini-batch (with zero mean and variance of one) in the hidden layers of the network. To alleviate the gradient internal covariate shift phenomenon and speed up the CNN training, an Adam Gradient Decent method was used to train the model (Kingma and Ba, 2015).

##### Activation layer

After the batch normalization operation, an activation function was used to non-linearize the convolution result. The activation function we used in the model was the Rectified linear unit, ReLU (Nair and Hinton, 2010).

##### Pooling layer

Pooling layer was added after the activation layer. Pooling layers in the CNN summarize the outputs of neighboring groups of neurons in the same kernel map (Krizhevsky et al., 2012). Max-pooling method was used in this layer.

The outputs of each hidden layer were feature maps, which were the features extracted from the input images to the hidden layer. The outputs from the previous hidden layer were the inputs to the next layer. In our model, the first hidden layer generated 32 feature maps, the second hidden layer produced 64 feature maps, and the third hidden layer yielded 128 feature maps. Finally, we integrated the last 128 feature maps into the input of the softmax layer through a linear layer, and then got the final classification results from the softmax layer.

In our model, the input *X* ∈ {*x*^(1)^, *x*^(2)^, …, *x*^(*n*)^}, *x*^(*i*)^ was the *ith* subject's FA value. *Y* ∈ {*y*^(1)^, *y*^(2)^, …, *y*^(*n*)^}, *y*^(*i*)^ was the *ith* subject's label that were processed to one-hot vector where [1 0] represents man and [0 1] woman. We used *h(*θ*, x)* to represent the proposed 3D PCNN model. Then we had:

(1) $$\begin{array}{r} ŷ=h\left(\theta ,x\right) \end{array}$$

where ŷ represents the predicted value obtained using the 3D PCNN on a sample x.

### Parameters Optimization

The initial values of the weights of the convolution kernels were random values selected from a truncated normal distribution with standard deviation of 0.1. We defined a cost function to adjust these weights based on the softmax cross entropy (Dunne and Campbell, 1997):

(2) $$\begin{array}{r} J\left(\theta ,x\right)=-\overset{n}{\underset{i=1}{\sum }}{ŷ}^{\left(i\right)}\log P\left({ŷ}^{\left(i\right)}=y^{\left(i\right)}\;|\;x=x^{\left(i\right)}\;\right) \end{array}$$

As such, the task of adjusting the weight value became an optimization problem with *J*(θ, *x*) as the optimization goal, where a small penalty was given if the classification result was correct, and vice versa. We used the Adam Gradient Descent (Kingma and Ba, 2015) optimization algorithm to achieve this goal in the model training. All parameters in the Adam algorithm were set to the empirical values recommended by Kingma and Ba (2015), i.e., learning rate was α = 0.001, exponential decay rates for the moment estimates were *β*_1_ = 0.9, *β*_1_ = 0.999, *ε* = 10^−8^.

### Cross-Validation

**Figure 2:**
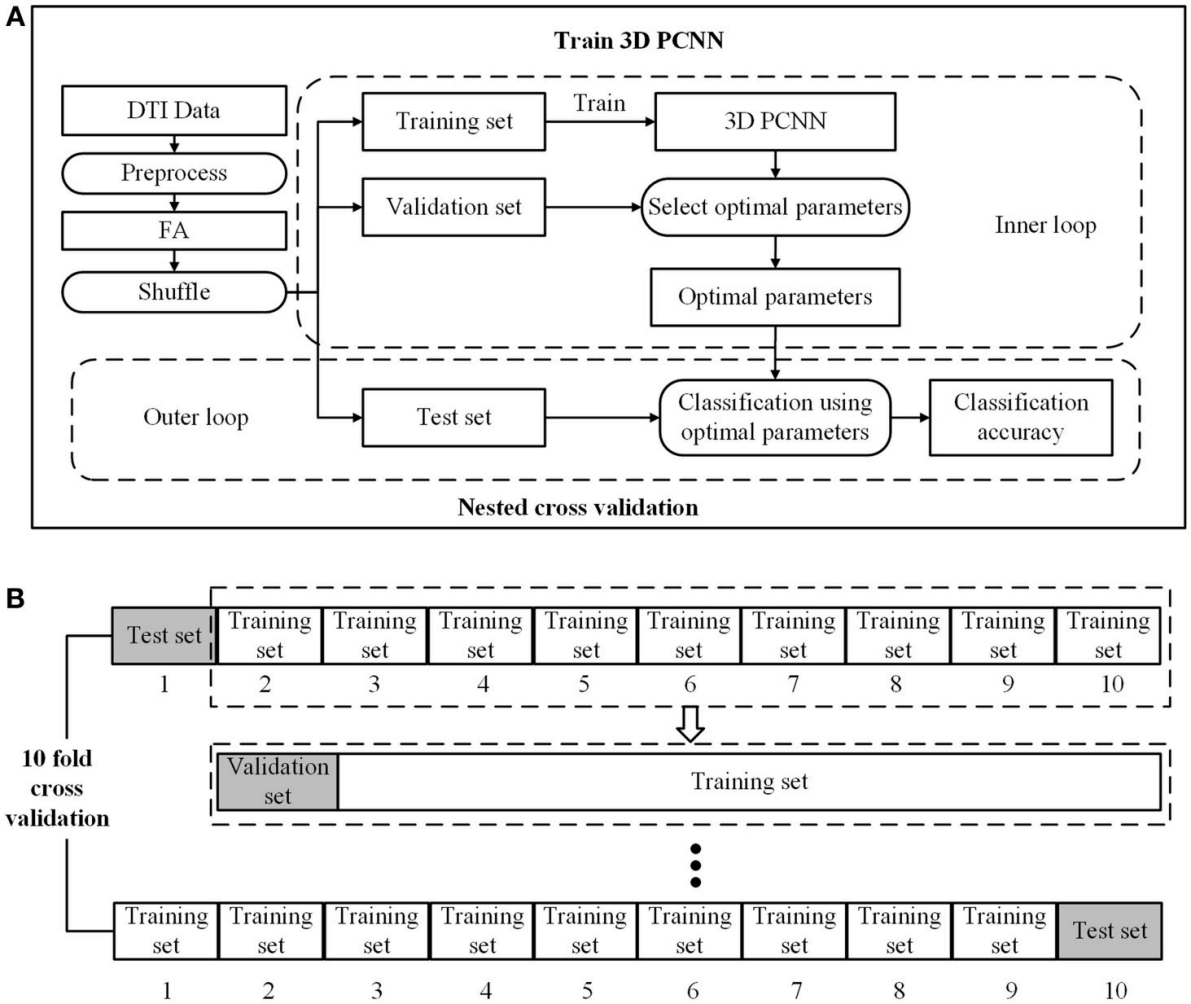
Model training and nested cross validation. **(A)** General overview. **(B)** 10 fold cross validation.

To ensure the independent training and testing in the cross-validation. The process of cross-validation is shown in Figure 2. We implemented a two-loop nested cross-validation scheme (Varoquaux et al., 2017). We divided the data set into three parts, i.e., 80% of the data as the training set for model training, 10% as the verification set for parameter selection, and 10% as the testing set for evaluating the generalization ability of the model. To eliminate the random error of model training, we run 10 fold cross validation and then took the average of classification accuracies as the final result.

### Features in First Hidden Layer

CNN has an advantage that it can extract key features by itself (Zeng et al., 2018c). However, these features may be difficult to interpret since they are highly abstract features. Thus, in this study, we only analyzed the features obtained in the first hidden layer, since they are the direct outputs from the convolution on the grayscale FA images. In this case, the convolution operation of the first layer is equivalent to applying a convolution kernel based spatial filter on the FA images. The obtained features are less abstractive than those from the second and three hidden layers. There are totally 32 features in the first hidden layer. These features are the lowest-level features which may represent the structural features of FA images. We firstly computed the mean of voxel values across all subjects in each group (man vs. woman) for each feature and then evaluated their group-level difference using a two-sample *t*-test. Besides, we also computed the entropy on each feature for each individual:

(3) $$\begin{array}{r} H=-\overset{255}{\underset{i=0}{\sum }}p_{i}\log p_{i} \end{array}$$

where *p*_*i*_ indicates the frequency of pixel with value *i* appears in the image. The entropy of each feature likely indicates the complexity of brain structural encoded in that feature. We also performed a two-sample *t*-test on entropy results to explore the differences between men and women. A strict Bonferroni correction was applied for multiple comparisons with the threshold of 0.05/32 = 1.56 × 10^−3^ to remove spurious significance.

### Discriminative Power of Brain Regions

In order to determine which brain regions may play important role in gender-related brain structural differences, we repeated the same 3D PCNN-based classification on each specific brain region. We segmented each FA image into 246 gray matter regions of interests (ROIs) according to the Human Brainnetome Atlas (Fan et al., 2016) and 48 white matter ROIs according to the ICBM-DTI-81 White-Matter Labels Atlas (Mori et al., 2005). The classification accuracy was then obtained for each ROIs. A higher accuracy indicates a more important role of that ROI in gender-related difference. A map was then obtained based on the classification accuracies of different ROIs to show their distribution in the brain.

### Comparisons With Tract Based Spatial Statistics and Support Vector Machine

To justify the effectiveness of our method, the Tract Based Spatial Statistics (TBSS) and Support Vector Machine (SVM) were applied to our dataset as comparisons, since these are two popular methods for data analysis in neuroimaging studies (Bach et al., 2014; Zeng et al., 2018b). We compared the results in following two conditions: (1) We used the SVM as the classifier while keeping the same preprocessing procedure in order to compare its results with our 3D PCNN method. We flatten each sample from the 3D FA matrix into a vector, and then fed the SVM with the vector. (2) We used the TBSS to identify the brain regions where are shown the statistically significant gender-related difference.

## Results

### Classification Results on the Whole-Brain FA Images

Using our 3D PCNN methods on the whole-brain FA images, we can well-distinguish men and women with the classification accuracy of 93.3%. This result is much better than using the SVM, whose classification accuracy is only 78.2%.

As comparisons, we also used MD, AD, and RD to repeat the same analysis. The classification accuracy of MD is 65.8%, AD is 69.9%, and RD is 67.8%. All of them are lower than the classification accuracy obtained by using FA.

### Feature Analysis in the First Hidden Layer of 3D PCNN

**Figure 3:**
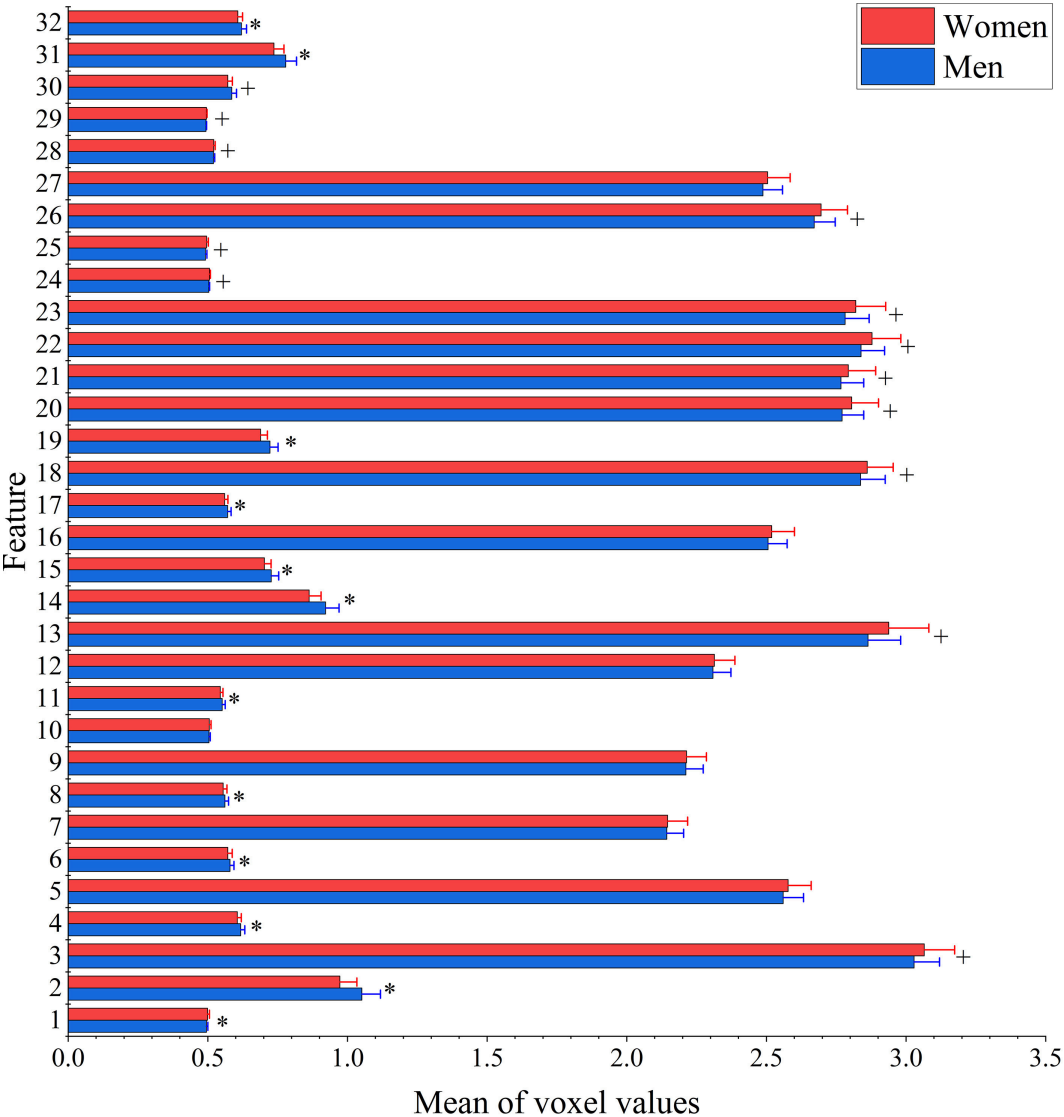
Between-group differences of 32 features in voxel values. The mean (bar height) and standard deviation (error bars) of voxel values across all subjects in each group were evaluated for each feature. Their group-level difference was examined using a two-sample *t*-test. Bonferroni correction was applied for multiple comparisons with the threshold equal to 0.05/32 = 1.56 × 10^−3^ to remove spurious significance. The features with significantly larger mean voxel values for men are marked out with*, while features with significantly larger mean voxel values for women are indicated by +.

**Figure 4:**
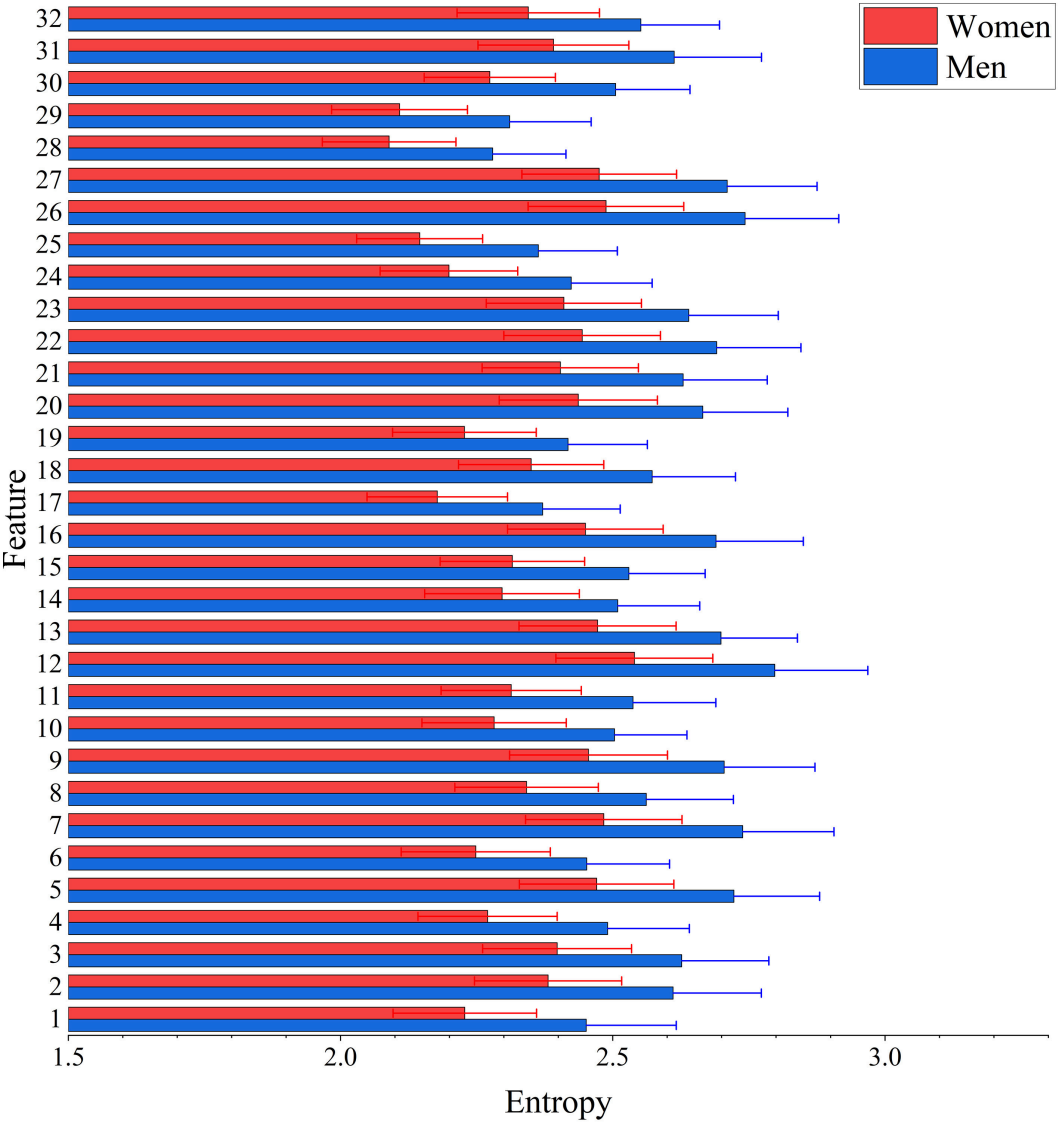
Between-group differences of 32 features in entropy values. The mean (bar height) and standard deviation (error bars) of entropy value were computed across all subjects in each group for each feature. Their group-level difference was evaulated using a two-sample *t*-test. Bonferroni correction was applied for multiple comparisons with the threshold equal to 0.05/32 = 1.56 × 10^−3^ to remove spurious significance. The entropy values are significantly larger in men than in women for features.

The result of two-sample *t*-test of 32 features of men and women shows that there are 25 features had significant gender differences including 13 features that women have larger values and 12 features that men have larger values (see Figure 3). Interestingly, men have significantly higher entropy than women for all features (see Figure 4).

### Classification on Each Specific ROI

**Figure 5:**
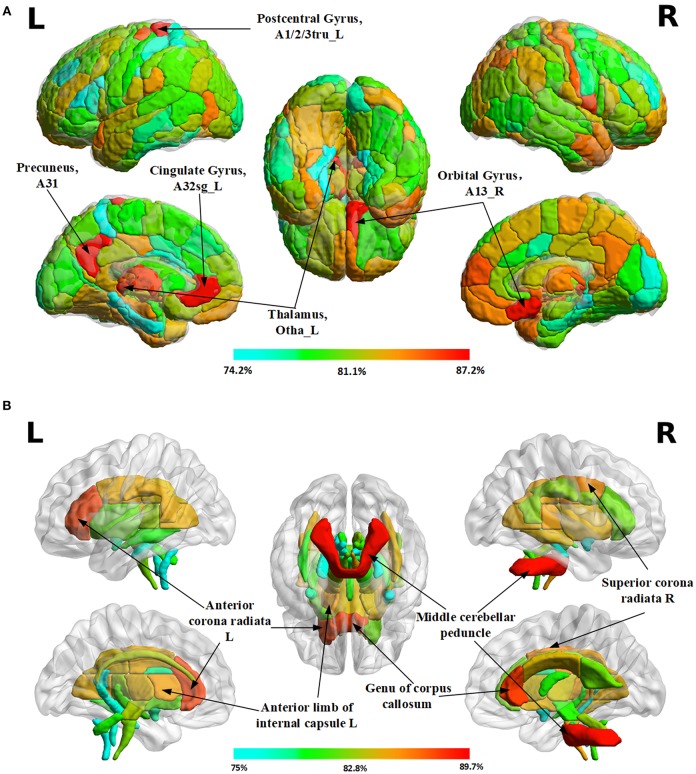
Maps of classification accuracies for different ROIs in the gray and white matter of the brain. **(A)** Results in 246 gray matter regions of interests (ROIs) according to the Human Brainnetome Atlas **(B)** Results in 48 white matter ROIs according to the ICBM-DTI-81 White-Matter Labels Atlas.

TBSS could not detect any statistically significant gender-related difference in this dataset. However, using 3D PCNN, we did find gender-related differences in all ROIs in the both gray and white matters, as the classification accuracies (>75%) are much higher than the chance level (50%) for all ROIs. The maps of classification accuracies for different ROIs are shown in Figure 5. The detail classification results are provided in the supplement (see Table S1 for gray matter and Table S2 for white matter). In the gray matter, the top 5 regions with highest classification accuracies are the left precuneus (Broadman area, BA 31, 87.2%), the left postcentral gyrus (BA 1/2/3 trunk region, 87.2%), the left cingulate gyrus (BA 32 subgenual area, 87.2%), the right orbital gyrus of frontal lobe (BA 13, 87.1%) and the left occipital thalamus (86.9%). In the white matter, the top 5 regions with highest classification accuracies are middle cerebellum peduncle (89.7%), genu of corpus callosum (88.4%), the right anterior corona radiata (88.3%), the right superior corona radiata (86%), and the left anterior limb of internal capsule (85.4%).

## Discussions

### Classification on the Whole-Brain FA

The proposed 3D PCNN model achieved 93.3% classification accuracy in the whole-brain FA. The high classification accuracy rate indicates that the proposed model can accurately find the brain structure difference between men and women, which is the basis of subsequent feature analysis and subreginal analysis. Most existing classification, regression, and other machine learning methods are shallow learning algorithms, such as the SVM, Boosting, maximum entropy, and Logistic Regression. When complex functions need to be expressed, the models obtained by these algorithms will then have a limitation with small size of samples and limited computational resources. Thus, the generalization ability will be deteriorated as we demonstrated in the results from the SVM. The benefit of deep learning algorithms, using multiple layers in the artificial neural network, is that one can represent complex functions with few parameters. The CNN is one of the widely used deep learning algorithms. In compared to the method like SVM, which is just a classifier, 3D CNN is a method that can extract the 3D spatial structure features of the input image. Through constructing the 3D PCNN model, we extracted highly abstract features from FA images, which may, thusly, improve the classification accuracy. FA describes the partial anisotropy index, which indicates the difference between one direction and others (Feldman et al., 2010). It can reflect alterations in various tissue properties including axonal size, axonal packing density, and degree of myelination (Chung et al., 2016). In this study, we also run the same analysis using MD, AD, and RD images for comparisons. All their results are lower than that of FA, indicating that using FA is more effective to find the structure difference between men and women's brain than using other images.

### Feature Analysis in the First Hidden Layer of 3D PCNN

The degree of the macroscopic diffusion anisotropy is often quantified by the FA (Lasi et al., 2014). Previous studies found that wider skeleton of white matter in woman's brain but wider region of gray matter in man's brain (Witelson et al., 1995; Zaidi, 2010; Gong et al., 2011; Menzler et al., 2011). These mean that men appear to have more gray matter, made up of active neurons, while women may have more white matter for the neuronal communication between different areas of the brain. Furthermore, a recent study found that men had higher FA values than women in middle aged to elderly (between 44 and 77 years old) people by using a statistical analysis (Ritchie et al., 2018). This study focuses on the young healthy individuals with the age range between 22 and 36 years old. The structural features extracted from 3D PCNN reflect the brain structure difference between men and women. In the first hidden layer of 3D PCNN model, we found 25 features that have significant difference between men and women in voxels value. Moreover, using entropy measure, we found that men's brains likely have more complex features as reflected by significantly higher entropy. These results indicated that the gender-related differences likely exist in the whole-brain range including both white and gray matters.

### Most Discriminative Brain Regions

Using FA images from each specific brain region as the input to the 3D PCNN, we found all tested brain regions may have gender-related difference, though the TBSS analysis cannot detect these differences. The brain regions with high classification accuracies include the left precuneus (Broadman area, BA 31, 87.2%), the left postcentral gyrus (BA 1/2/3 trunk region, 87.2%), the left cingulate gyrus (BA 32 subgenual area, 87.2%), the right orbital gyrus of frontal lobe (BA 13, 87.1%), and the left occipital thalamus (86.9%) in the gray matter, and middle cerebellum peduncle (89.7%), genu of corpus callosum (88.4%), the right anterior corona radiata (88.3%), the right superior corona radiata (86%), and the left anterior limb of internal capsule (85.4%).

The gender-related morphological difference at the corpus callosum has been previously reported, which may be associated with interhemispheric interaction (Sullivan et al., 2001; Luders et al., 2003; Prendergast et al., 2015). However, likely due to the limitation of applied methods, not all previous studies have reported this difference (Abe et al., 2002). Those likely results in the inconsistent findings were across different studies. Through 3D PCNN model, our results confirm that there is likely a morphological difference at the genu of corpus callosum between man and women.

The middle cerebellum peduncle is the brain area connected to the pons and receiving the inputs mainly from the pontine nuclei (Glickstein and Doron, 2008), which are the nuclei of the pons involved in motor activity (Wiesendanger et al., 1979). Raz et al. (2001) found larger volume in the cerebellum of men than women. The cerebellar cells release diffusible substances that promote the survival of thalamic neurons (Tracey et al., 1980; Hisanaga and Sharp, 1990). Previous studies have reported gender-difference differences in the basic glucose metabolism in the thalamus of young subjects between the ages of 20 and 40 (Fujimoto et al., 2008). Beside the thalamus and cerebellum, the postcentral gyrus was also found in our results as the brain region with high classification accuracy. Thus, there is very likely a gender-related difference in the cerebellar-thalamic-cortical circuitry. This difference may also be related to the reported gender differences in neurological degenerative diseases such as Parkinson's Disease (Lyons et al., 1998; Dluzen and Mcdermott, 2000; Miller and Cronin-Golomb, 2010), where the pathological changes are usually found in the cerebellar-thalamic-cortical circuitry.

The findings of the current study also indicated the gender-related difference in the limbic-thalamo-cortical circuitry. Anterior corona radiata is part of the limbic-thalamo-cortical circuitry and includes thalamic projections from the internal capsule to the prefrontal cortex. White matter changes in the anterior corona radiata could result in many of the cognitive and emotion regulation disturbances (Drevets, 2001). The orbital gyrus of frontal cortex gray matter areas and cingulate gyrus have also been reported to be associated with the emotion regulation system (Fan et al., 2005). Thus, the gender-related difference in the limbic-thalamo-cortical circuitry may explain the gender differences in thalamic activation during the processing of emotional stimuli or unpleasant linguistic information concerning interpersonal difficulties as demonstrated by previous fMRI (Lee and Kondziolka, 2005; Shirao et al., 2005).

In summary, by using the designed 3D PCNN algorithm, we confirmed that the gender-related differences exist in the whole-brain FA images as well as in each specific brain regions. These gender-related brain structural differences might be related to gender differences in cognition, emotional control as well as neurological disorders.

## Data Availability

Publicly available datasets were analyzed in this study. This data can be found here: https://www.humanconnectome.org/.

## Author Contributions

JX, YT, and YY contributed to the conception and design of the study. YT, JX, and YZ performed data analysis. YT and JX drafted manuscript. YT and YY participated in editing the manuscript.

### Conflict of Interest Statement

The authors declare that the research was conducted in the absence of any commercial or financial relationships that could be construed as a potential conflict of interest.
