# Supplementary material for: Brain Differences Between Men and Women: Evidence From Deep Learning
Source: Front Neurosci. 2019 Mar 8;13:185. doi: 10.3389/fnins.2019.00185 (PMC6418873; doi:10.3389/fnins.2019.00185)
Supplement: Supplementary file 1 [file Table_1.docx]

**Supplementary Material**

Brain Differences between Men and Women: Evidence from Deep Learning

Jiang Xin^1^, Yaoxue Zhang^1^, Yan Tang^1,2*^, Yuan Yang^3*^

^1^ School of Computer Science and Engineering, Central South University, Changsha, Hunan 410083, China.

^2^Department of Neurology, Xiangya Hospital, Central South University, Changsha, 410008 Hunan, China

^3^Department of Physical Therapy and Human Movement Sciences, Feinberg School of Medicine, Northwestern University, Chicago, IL 60611, USA

*** Co-correspondence:**Yan Tang, [Tangyan@csu.edu.cn](mailto:Tangyan@csu.edu.cn)
Yuan Yang, [yuan.yang@northwestern.edu](mailto:yuan.yang@northwestern.edu)

**Table S1** Classification accuracy (CA) of each brain region in the grey matter.

| Modified Cyto-architectonic | Label ID.L | CA | Label ID.R | CA |
| --- | --- | --- | --- | --- |
| A8m, medial area 8 | 1 | 79.3% | 2 | 82.4% |
| A8dl, dorsolateral area 8 | 3 | 81.5% | 4 | 82.0% |
| A9l, lateral area 9 | 5 | 79.8% | 6 | 79.4% |
| A6dl, dorsolateral area 6 | 7 | 80.9% | 8 | 77.6% |
| A6m, medial area 6 | 9 | 80.4% | 10 | 80.7% |
| A9m,medial area 9 | 11 | 77.8% | 12 | 82.6% |
| A10m, medial area 10 | 13 | 81.0% | 14 | 85.4% |
| A9/46d, dorsal area 9/46 | 15 | 76.1% | 16 | 77.4% |
| IFJ, inferior frontal junction | 17 | 81.9% | 18 | 78.7% |
| A46, area 46 | 19 | 80.9% | 20 | 76.2% |
| A9/46v, ventral area 9/46 | 21 | 82.4% | 22 | 81.1% |
| A8vl, ventrolateral area 8 | 23 | 79.9% | 24 | 78.9% |
| A6vl, ventrolateral area 6 | 25 | 81.9% | 26 | 78.5% |
| A10l, lateral area10 | 27 | 81.4% | 28 | 82.8% |
| A44d,dorsal area 44 | 29 | 75.7% | 30 | 82.1% |
| IFS, inferior frontal sulcus | 31 | 75.4% | 32 | 77.4% |
| A45c, caudal area 45 | 33 | 76.5% | 34 | 82.2% |
| A45r, rostral area 45 | 35 | 78.9% | 36 | 80.9% |
| A44op, opercular area 44 | 37 | 82.3% | 38 | 81.1% |
| A44v, ventral area 44 | 39 | 81.5% | 40 | 79.9% |
| A14m, medial area 14 | 41 | 82.1% | 42 | 84.4% |
| A12/47o, orbital area 12/47 | 43 | 79.0% | 44 | 79.3% |
| A11l, lateral area 11 | 45 | 81.6% | 46 | 79.3% |
| A11m, medial area 11 | 47 | 83.3% | 48 | 83.9% |
| A13, area 13 | 49 | 81.3% | 50 | 87.1% |
| A12/47l, lateral area 12/47 | 51 | 79.5% | 52 | 81.1% |
| A4hf, area 4(head and face region) | 53 | 80.0% | 54 | 77.7% |
| A6cdl, caudal dorsolateral area 6 | 55 | 79.7% | 56 | 78.5% |
| A4ul, area 4(upper limb region) | 57 | 85.5% | 58 | 84.1% |
| A4t, area 4(trunk region) | 59 | 80.3% | 60 | 84.0% |
| A4tl, area 4(tongue and larynx region) | 61 | 79.0% | 62 | 86.1% |
| A6cvl, caudal ventrolateral area 6 | 63 | 79.4% | 64 | 78.5% |
| A1/2/3ll, area1/2/3 (lower limb region) | 65 | 78.9% | 66 | 82.0% |
| A4ll, area 4, (lower limb region) | 67 | 78.3% | 68 | 83.3% |
| A38m, medial area 38 | 69 | 82.5% | 70 | 84.4% |
| A41/42, area 41/42 | 71 | 79.2% | 72 | 80.0% |
| TE1.0 and TE1.2 | 73 | 82.7% | 74 | 82.0% |
| A22c, caudal area 22 | 75 | 79.7% | 76 | 80.2% |
| A38l, lateral area 38 | 77 | 84.3% | 78 | 83.8% |
| A22r, rostral area 22 | 79 | 82.0% | 80 | 79.2% |
| A21c, caudal area 21 | 81 | 77.7% | 82 | 80.2% |
| A21r, rostral area 21 | 83 | 82.0% | 84 | 84.5% |
| A37dl, dorsolateral area37 | 85 | 79.3% | 86 | 78.5% |
| aSTS, anterior superior temporal sulcus | 87 | 81.3% | 88 | 80.2% |
| A20iv, intermediate ventral area 20 | 89 | 81.8% | 90 | 80.8% |
| A37elv, extreme lateroventral area37 | 91 | 77.3% | 92 | 83.3% |
| A20r, rostral area 20 | 93 | 80.5% | 94 | 82.0% |
| A20il, intermediate lateral area 20 | 95 | 79.3% | 96 | 78.9% |
| A37vl, ventrolateral area 37 | 97 | 81.5% | 98 | 77.8% |
| A20cl, caudolateral of area 20 | 99 | 80.8% | 100 | 79.8% |
| A20cv, caudoventral of area 20 | 101 | 77.4% | 102 | 79.8% |
| A20rv, rostroventral area 20 | 103 | 83.7% | 104 | 80.2% |
| A37mv, medioventral area37 | 105 | 82.8% | 106 | 78.2% |
| A37lv, lateroventral area37 | 107 | 82.6% | 108 | 79.4% |
| A35/36r, rostral area 35/36 | 109 | 83.1% | 110 | 77.0% |
| A35/36c, caudal area 35/36 | 111 | 76.0% | 112 | 77.4% |
| TL, area TL (lateral PPHC, posterior parahippocampal gyrus) | 113 | 78.2% | 114 | 77.6% |
| A28/34, area 28/34 (EC, entorhinal cortex) | 115 | 75.6% | 116 | 77.2% |
| TI, area TI(temporal agranular insular cortex) | 117 | 78.1% | 118 | 80.1% |
| TH, area TH (medial PPHC) | 119 | 75.6% | 120 | 74.2% |
| rpSTS, rostroposterior superior temporal sulcus | 121 | 81.9% | 122 | 77.9% |
| cpSTS, caudoposterior superior temporal sulcus | 123 | 81.8% | 124 | 82.5% |
| A7r, rostral area 7 | 125 | 81.3% | 126 | 80.8% |
| A7c, caudal area 7 | 127 | 80.1% | 128 | 82.9% |
| A5l, lateral area 5 | 129 | 76.2% | 130 | 78.2% |
| A7pc, postcentral area 7 | 131 | 76.5% | 132 | 78.9% |
| A7ip, intraparietal area 7(hIP3) | 133 | 78.7% | 134 | 79.2% |
| A39c, caudal area 39(PGp) | 135 | 80.3% | 136 | 80.8% |
| A39rd, rostrodorsal area 39(Hip3) | 137 | 81.1% | 138 | 84.4% |
| A40rd, rostrodorsal area 40(PFt) | 139 | 80.5% | 140 | 81.1% |
| A40c, caudal area 40(PFm) | 141 | 79.9% | 142 | 82.6% |
| A39rv, rostroventral area 39(PGa) | 143 | 81.7% | 144 | 80.8% |
| A40rv, rostroventral area 40(PFop) | 145 | 83.1% | 146 | 79.6% |
| A7m, medial area 7(PEp) | 147 | 80.0% | 148 | 82.7% |
| A5m, medial area 5(PEm) | 149 | 75.0% | 150 | 82.6% |
| dmPOS, dorsomedial parietooccipital sulcus(PEr) | 151 | 82.1% | 152 | 83.6% |
| A31, area 31 (Lc1) | 153 | 87.2% | 154 | 84.2% |
| A1/2/3ulhf, area 1/2/3(upper limb, head and face region) | 155 | 78.4% | 156 | 85.1% |
| A1/2/3tonLa, area 1/2/3(tongue and larynx region) | 157 | 82.0% | 158 | 82.6% |
| A2, area 2 | 159 | 76.6% | 160 | 81.1% |
| A1/2/3tru, area1/2/3(trunk region) | 161 | 87.2% | 162 | 82.9% |
| G, hypergranular insula | 163 | 82.4% | 164 | 83.4% |
| vIa, ventral agranular insula | 165 | 82.7% | 166 | 79.8% |
| dIa, dorsal agranular insula | 167 | 81.7% | 168 | 76.9% |
| vId/vIg, ventral dysgranular and granular insula | 169 | 79.6% | 170 | 82.5% |
| dIg, dorsal granular insula | 171 | 78.0% | 172 | 81.0% |
| dId, dorsal dysgranular insula | 173 | 83.2% | 174 | 79.5% |
| A23d, dorsal area 23 | 175 | 81.5% | 176 | 82.0% |
| A24rv, rostroventral area 24 | 177 | 76.5% | 178 | 80.2% |
| A32p, pregenual area 32 | 179 | 79.0% | 180 | 78.2% |
| A23v, ventral area 23 | 181 | 82. 6% | 182 | 83.8% |
| A24cd, caudodorsal area 24 | 183 | 77.6% | 184 | 77.2% |
| A23c, caudal area 23 | 185 | 83.4% | 186 | 81.9% |
| A32sg, subgenual area 32 | 187 | 87. 2% | 188 | 81.2% |
| cLinG, caudal lingual gyrus | 189 | 81.2% | 190 | 76.9% |
| rCunG, rostral cuneus gyrus | 191 | 78.7% | 192 | 78.6% |
| cCunG, caudal cuneus gyrus | 193 | 79.4% | 194 | 76.0% |
| rLinG, rostral lingual gyrus | 195 | 82.7% | 196 | 80.5% |
| vmPOS, ventromedial parietooccipital sulcus | 197 | 77.9% | 198 | 82.6% |
| mOccG, middle occipital gyrus | 199 | 80.6% | 200 | 82.4% |
| V5/MT+, area V5/MT+ | 201 | 84.2% | 202 | 78.3% |
| OPC, occipital polar cortex | 203 | 77.6% | 204 | 79.9% |
| iOccG, inferior occipital gyrus | 205 | 81.3% | 206 | 82.8% |
| msOccG, medial superior occipital gyrus | 207 | 80.2% | 208 | 84.1% |
| lsOccG, lateral superior occipital gyrus | 209 | 82.6% | 210 | 82.9% |
| mAmyg, medial amygdala | 211 | 79.0% | 212 | 81.1% |
| lAmyg, lateral amygdala | 213 | 79.5% | 214 | 85.2% |
| rHipp, rostral hippocampus | 215 | 83.7% | 216 | 80.9% |
| cHipp, caudal hippocampus | 217 | 82.4% | 218 | 76.9% |
| vCa, ventral caudate | 219 | 78.5% | 220 | 82.3% |
| GP, globus pallidus | 221 | 84. 1% | 222 | 81.1% |
| NAC, nucleus accumbens | 223 | 84.4% | 224 | 85.0% |
| vmPu, ventromedial putamen | 225 | 84.7% | 226 | 85.4% |
| dCa, dorsal caudate | 227 | 79.8% | 228 | 81.9% |
| dlPu, dorsolateral putamen | 229 | 83.7% | 230 | 83.3% |
| mPFtha, medial pre-frontal thalamus | 231 | 86.1% | 232 | 81.0% |
| mPMtha, pre-motor thalamus | 233 | 83.2% | 234 | 81.4% |
| Stha, sensory thalamus | 235 | 79.7% | 236 | 83.7% |
| rTtha, rostral temporal thalamus | 237 | 86.1% | 238 | 83.9% |
| PPtha, posterior parietal thalamus | 239 | 80.8% | 240 | 86.1% |
| Otha, occipital thalamus | 241 | 86.9% | 242 | 85.1% |
| cTtha, caudal temporal thalamus | 243 | 84.9% | 244 | 84.2% |
| lPFtha, lateral pre-frontal thalamus | 245 | 82.2% | 246 | 82.7% |

Table S2 Classification accuracy (CA) of each brain region in the white matter.

| Modified Cyto-architectonic | Label ID | CA | Modified Cyto-architectonic | Label ID | CA |
| --- | --- | --- | --- | --- | --- |
| Middle cerebellar peduncle | 1 | 89.7% | Pontine crossing tract (a part of MCP) | 2 | 79.2% |
| Genu of corpus callosum | 3 | 88.4% | Body of corpus callosum | 4 | 84.2% |
| Splenium of corpus callosum | 5 | 82.0% | Fornix (column and body of fornix) | 6 | 80.4% |
| Corticospinal tract R | 7 | 85.0% | Corticospinal tract L | 8 | 82.2% |
| Medial lemniscus R | 9 | 75.0% | Medial lemniscus L | 10 | 77.2% |
| Inferior cerebellar peduncle R | 11 | 81.0% | Inferior cerebellar peduncle L | 12 | 75.1% |
| Superior cerebellar peduncle R | 13 | 82.7% | Superior cerebellar peduncle L | 14 | 80.4% |
| Cerebral peduncle R | 15 | 80.7% | Cerebral peduncle L | 16 | 83.1% |
| Anterior limb of internal capsule R | 17 | 84.1% | Anterior limb of internal capsule L | 18 | 85.4% |
| Posterior limb of internal capsule R | 19 | 83.8% | Posterior limb of internal capsule L | 20 | 84.5% |
| Retrolenticular part of internal capsule R | 21 | 85.3% | Retrolenticular part of internal capsule L | 22 | 82.8% |
| Anterior corona radiata R | 23 | 81.9% | Anterior corona radiata L | 24 | 88.3% |
| Superior corona radiata R | 25 | 86.0% | Superior corona radiata L | 26 | 84.8% |
| Posterior corona radiata R | 27 | 85.0% | Posterior corona radiata L | 28 | 84.2% |
| Posterior thalamic radiation (include optic radiation) R | 29 | 84.7% | Posterior thalamic radiation (include optic radiation) L | 30 | 84.6% |
| inferior fronto-occipital fasciculus) R | 31 | 84.4% | Sagittal stratum (include inferior longitidinal fasciculus and inferior fronto-occipital fasciculus) L | 32 | 80.2% |
| External capsule R | 33 | 84.5% | External capsule L | 34 | 81.7% |
| Cingulum (cingulate gyrus) R | 35 | 82.4% | Cinguum (cingulate gyrus) L | 36 | 83.3% |
| Cingulum (hippocampus) R | 37 | 76.9% | Cingulum (hippocampus) L | 38 | 77.0% |
| Fornix (cres) / Stria terminalis R | 39 | 80.1% | Fornix (cres) / Stria terminalis L | 40 | 81.1% |
| Superior longitudinal fasciculus R | 41 | 82.5% | Superior longitudinal fasciculus L | 42 | 84.8% |
| Superior fronto-occipital fasciculus (could be a part of anterior internal capsule) R | 43 | 78.7% | Superior fronto-occipital fasciculus (could be a part of anterior internal capsule) L | 44 | 78.8% |
| Uncinate fasciculus R | 45 | 75.3% | Uncinate fasciculus L | 46 | 78.5% |
| Tapetum R | 47 | 84.0% | Tapetum L | 48 | 75.2% |
